# Familial Cleidocranial Dysplasia

**DOI:** 10.5005/jp-journals-10005-1055

**Published:** 2010-04-15

**Authors:** Radhika Verma, MK Jindal, Sandhya Maheshwari

**Affiliations:** 1 Tutor, Department of Pedodontics, Faculty of Medicine, Aligarh Muslim University, Aligarh, Uttar Pradesh, India; 2 Chairman and Associate Professor, Department of Pedodontics, Faculty of Medicine, Aligarh Muslim University, Aligarh Uttar Pradesh, India; 3 Professor, Department of Orthodontics, Faculty of Medicine, Aligarh Muslim University, Aligarh, Uttar Pradesh, India

**Keywords:** Cleidocranial dysplasia, autosomal dominant, skeletal dysplasia, retained primary, delayed eruption, partial anodontia.

## Abstract

Cleidocranial dysplasia or mutational dysostosis or cleidocranial dysostosis is a generalized skeletodental dysplastic disorder, inherited in an autosomal dominant pattern. The expression of this disorder can vary widely in severity, even within the same family. This is a relatively rare disease and has no standard effective treatment option as of yet. Here we present a case report of affected mother and son with classical manifestations of the disease.

## INTRODUCTION

The term cleidocranial dysplasia was coined in 1765 by Martin. ^1^ The thorough description of this disorder is attributed to Marie and Sainton in 1898^2^ and the disease is therefore also referred to as Marie-Saintons disease. This disease is known to have an autosomal dominant pattern of inheritance. Cleidocranial dysplasia is listed as a “rare disease” by the Office of Rare Diseases (ORD) of the National Institutes of Health (NIH). This means that cleidocranial dysplasia, or a subtype of cleidocranial dysplasia occurs in less than 200,000 individuals across the globe or approximately 1 per million affected individuals worldwide.

The RUNX2 gene provides instructions for making a protein that is involved in bone and cartilage development and maintenance.^3^ This ’transcription factor’ reportedly regulates a number of other genes needed for the optimum function of the osteoblasts. A change in one of the alleles of this gene; whether point mutation or deletion; interferes with normal bone and cartilage development, resulting in the signs and symptoms of cleidocranial dysplasia.

Familiar components of cleidocranial dysostosis include malformations of the hard tissues of the body specially the cranium, the clavicle and the dental tissues the malformations of the skull include brachycephaly with wide interparietal diameter, hypertelorism flat nasal bridge and hypoplastic upper jaw, leading to a flat faced look,^4^ open metopic fontanelle and suture numerous wormian bones and delayed suture closure.^5^ In addition, segmental calvarial thickening has been reported specially in the supraorbital portion of the frontal bone, in the squama of the temporal bones and in the occipital bone above the inion, producing overlying eminences. Dysplastic changes of basi-occiput leading to deformity of the foramen magnum are often seen as well. ^6^

Marked sclerosis and thickening of the frontal bone, squamosal portion of the temporal bones is also seen. Also, the squama changes from its vertical orientation into somewhat of an oblique position.^6^

Obvious clavicular dysplasia is one of consistent features of the disease. Either the clavicles fail to develop or are partially present. These patients can therefore characteristically easily get their shoulders to appose in the midline.^5,6^

Spine with hemivertebrae, spondylosis, a cone shaped thorax, hypoplasia and delayed fusion of pelvis, broad short hands with hypoplastic phalanges are the other skeletal findings. The skeletal abnormalities can make the patient shorter than normal, scoliotic and knock-kneed.^4^

Dental dysplasia occurs in almost all patients manifested mainly by delayed eruption or failure of eruption of permanent dentition and by supernumerary teeth. The permanent teeth either fail to erupt or do so gradually one by one in late adulthood.^4-6^

## CASE REPORT

A 9-year-old boy presented to the clinics of Department of Pedodontics, Dr Ziauddin Ahmed Dental College and Hospital, Aligarh Muslim University, Aligarh, with his mother. The mother stated that none of his milk teeth had yet shed.

The cause of her concern was that she herself had faced the same condition as a child and due to lack of awareness and funds, had not had a chance to rectify the situation. At present at the age of 32 she wore over dentures.

**Fig. 1:**
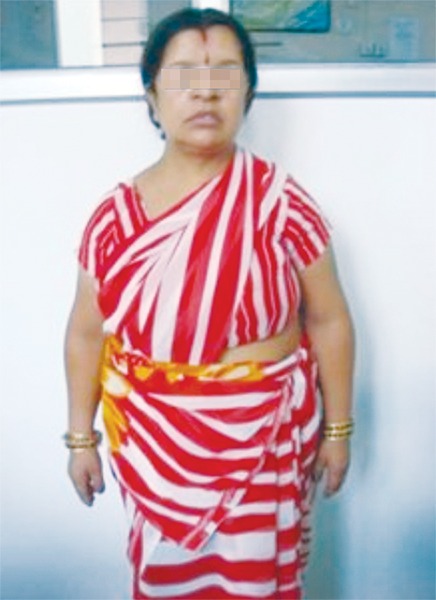
Stature of the mother of the 9 years old affected by cleidocranial dysplasia

**Fig. 2:**
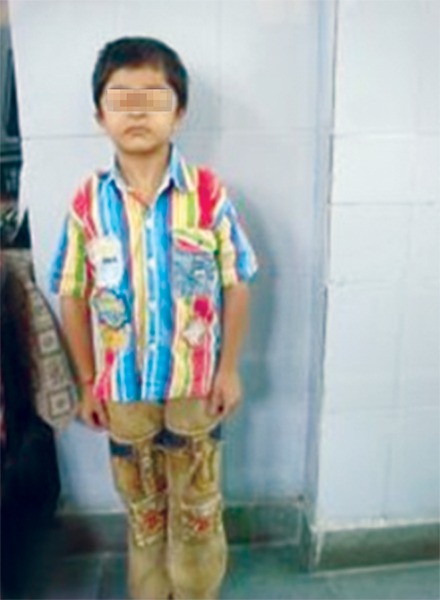
Stature of the 9 years old male affected by cleidocra-nial dysplasia

Upon observation, the facies and the stance of both the mother and son were suggestive of the classical manifestations of cleidocranial dysplasia (Figs 1 and 2).

**Fig. 3:**
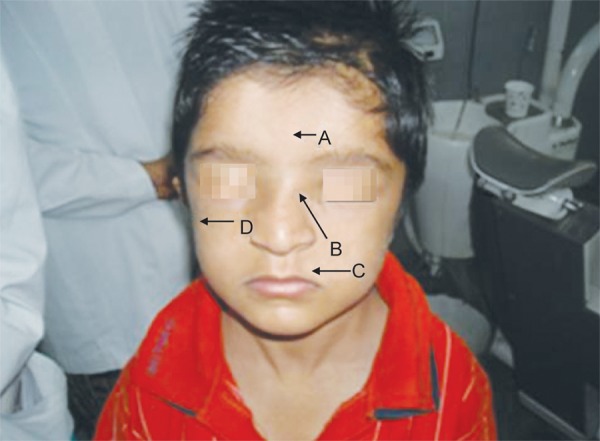
The 9-year old male affected by cleidocranial dysplasia (A. Depressed forehead with frontal bossing; B. Flat nasal bridge; C. Hypoplastic maxilla; D. Low set ears)

The child (Fig. 3) had a short stature, flat nasal bridge, widely spaced eyes, a malformed head and short and broad hands. He had broad neck, sloping and hypermobile shoulders.

His physical development, intelligence, hearing, spine and rest of the systemic examination were normal.

**Fig. 4:**
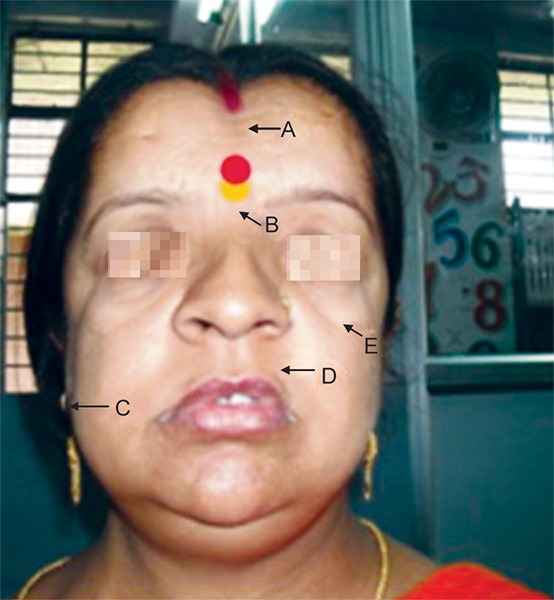
The mother (32-year-old) of the child (our case) also affected by cleidocranial dysplasia showing (A. Depressed forehead with frontal bossing; B. Flat nasal bridge; C. Hypoplastic maxilla; D Low set ears; E. Depressed malar bones

**Fig. 5:**
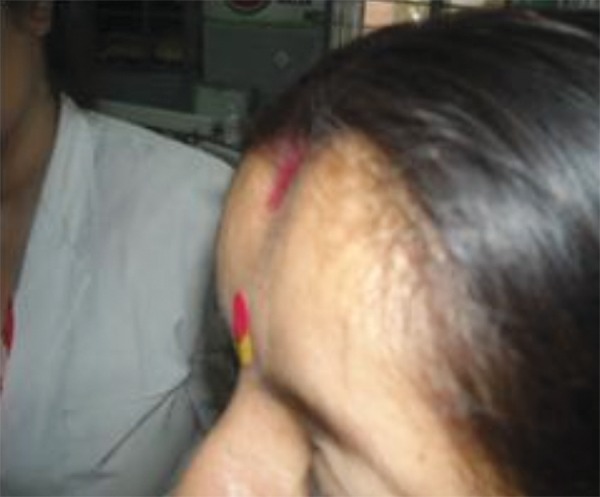
Depressed forehead with frontal bossing in mother

In the mother’s case (Figs 4 and 5), the skeletal features well as the degree of apposition of the shoulders were more pronounced.

**Fig. 6:**
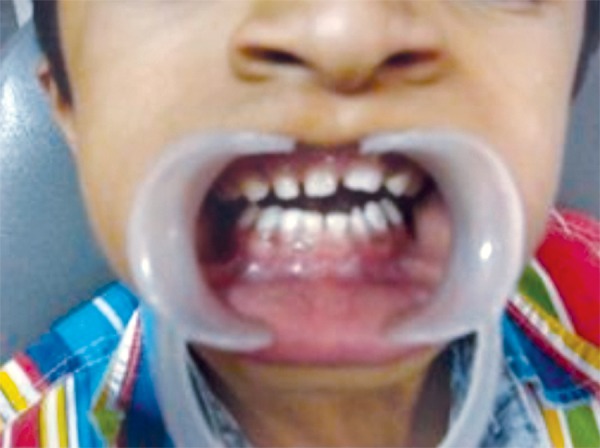
Intraoral photograph of the 9 years old male affected by cleidocranial dysplasia

**Fig. 7:**
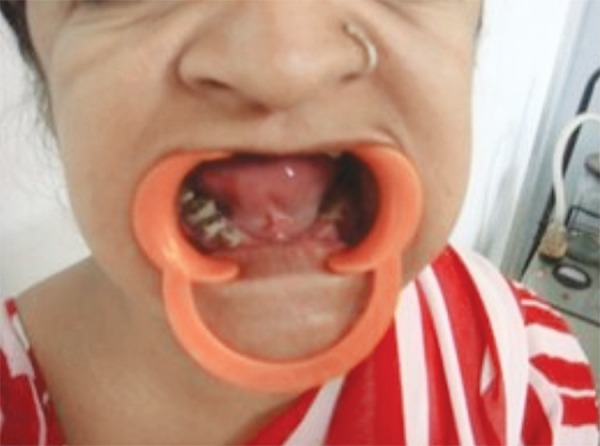
Intraoral photograph of the mother of our patient (also affected by cleidocranial dysplasia)

Clinical examination of the 9-year-old male revealed a high arched palate and malar and maxillary hypoplasia as well as fused upper left primary central and lateral incisor. There was an absence of spacing in the lower jaw (Fig. 6). None of this child’s teeth were mobile. The mother had partial anodontia with some erupted /malformed permanent teeth and mobile deciduous teeth present (Fig. 7).

**Fig. 8:**
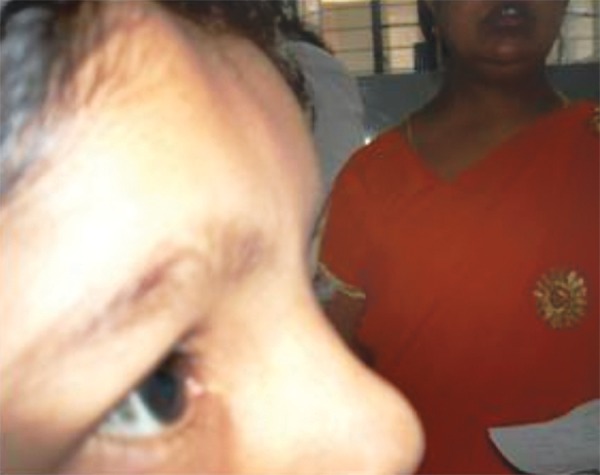
Depressed forehead with frontal bossing in the child

The head circumference was 50.5 cm (brachycephalic), with coronal suture ridging, depressed metopic suture (Fig. 8), just like in the mothers case (Fig. 5), hypertelorism and depressed nasal bridge. His ears were set low (Fig. 3).

**Fig. 9:**
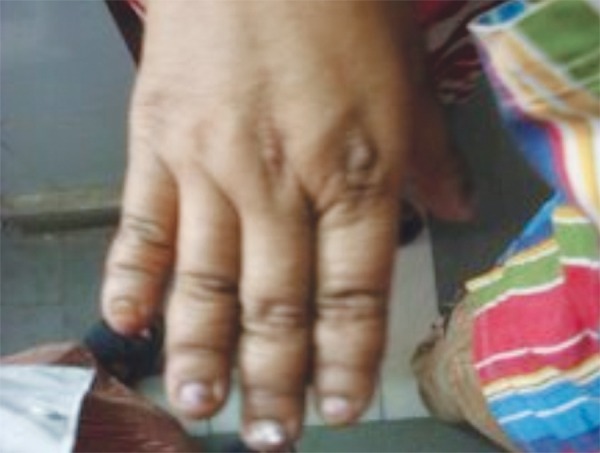
Hands of the mother of our patient (also affected by cleidocranial dysplasia), showing brachydactyly

**Fig. 10:**
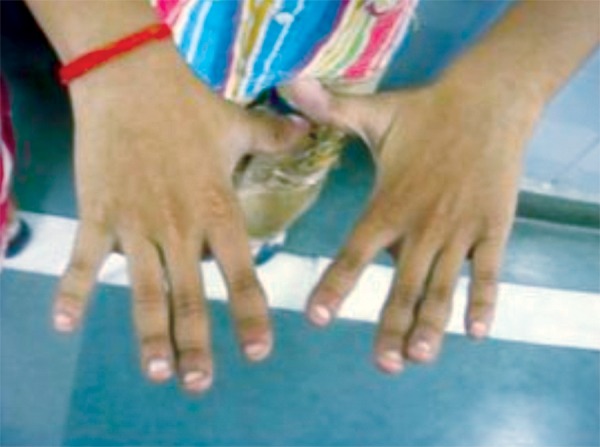
Hands of the 9 years old male affected by cleidocranial dysplasia

Upon palpation, the anterior fontanelle was judged to be not fully closed and clavicles were judged to be laterally deficient. The mother had more pronounced brachydactyly (Fig. 9) than the son (Fig. 10).

**Fig. 11:**
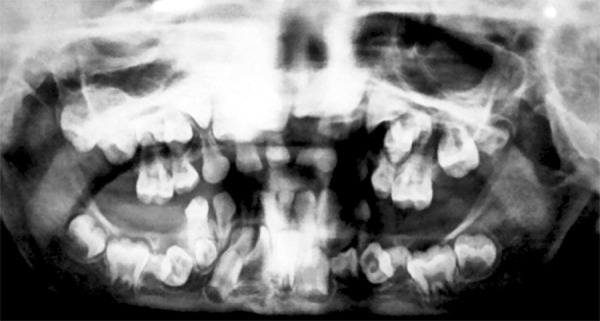
Orthopentamogram of the 9 years old male with cleido-cranial dysplasia showing deciduous retained teeth, numerous supernumerary teeth and dental age lagging behind chronological age

**Fig. 12:**
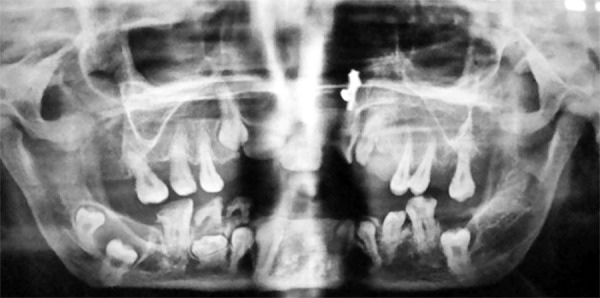
Orthopentamogram of the mother (32 years) of the pediatric case of cleidocranial dysplasia (9 years), also affected with this condition showing deciduous retained teeth, poorly formed permanent teeth and impacted permanent teeth with supernumerary teeth and dental age lagging behind chronological age

An OPG and X-ray chest were advised. The OPG showed that most of the permanent teeth had formed crowns but the root formation was lagging behind (Fig. 11). An OPG was also requested of the mother and it showed deciduous retained teeth, poorly formed permanent teeth and impacted permanent teeth with supernumerary teeth and dental age lagging behind chronological age (Fig. 12).

**Fig. 13:**
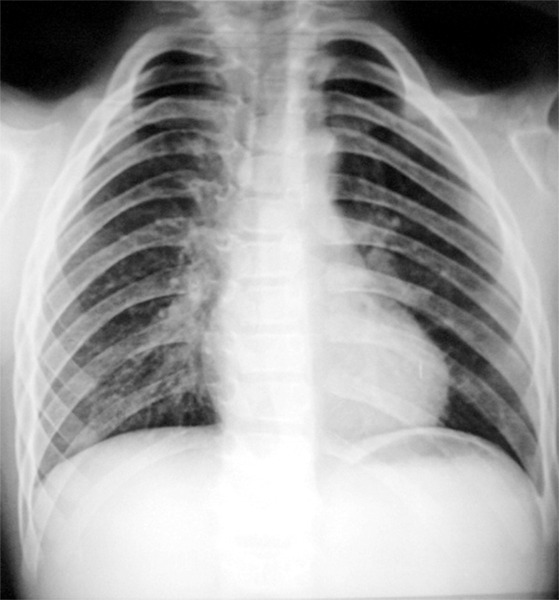
X-ray PA view chest of the 9-year-old male with cleidocranial dysplasia, showing laterally deficient clavicles

The X- ray chest showed clavicles were deficient laterally (Fig.13).

**Fig. 14:**
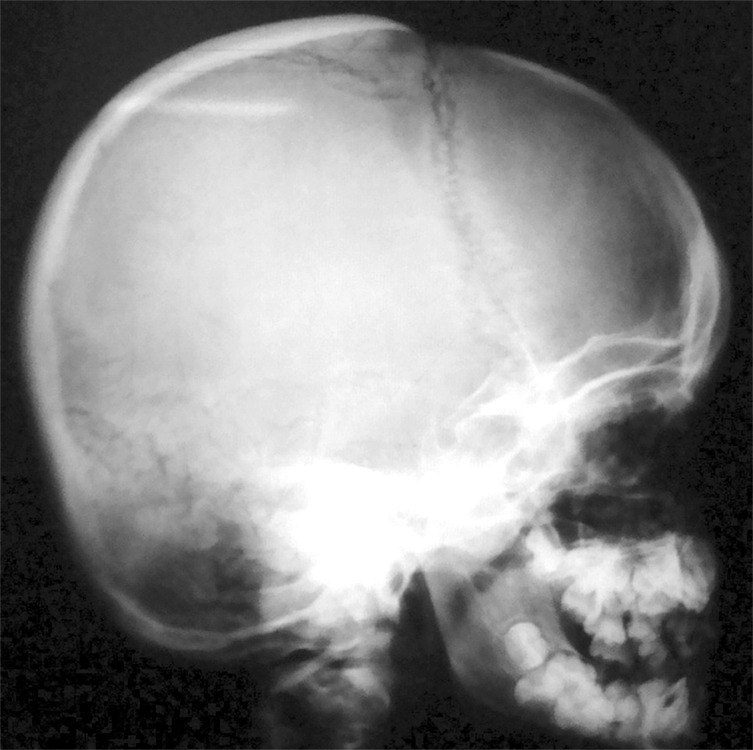
Lateral cephalogram of 9 years old male with cleido-cranial dysplasia, showing parietal frontal and occipital bossing

**Fig. 15:**
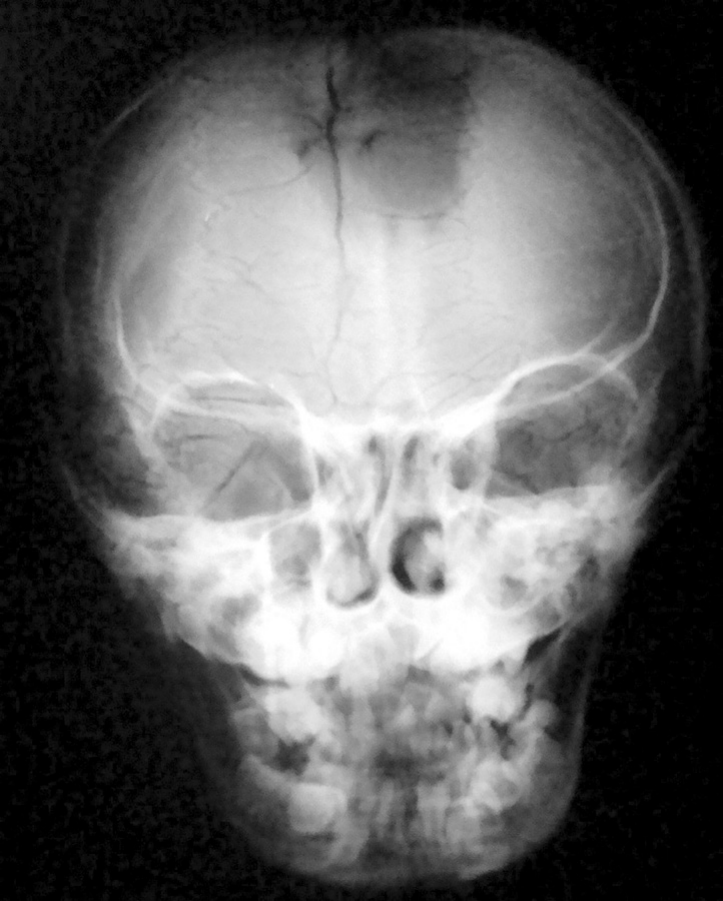
PA view head of 9 years old male with cleidocranial dysplasia showing open metopic fontanelle and suture with numerous Wormian bones

**Fig. 16:**
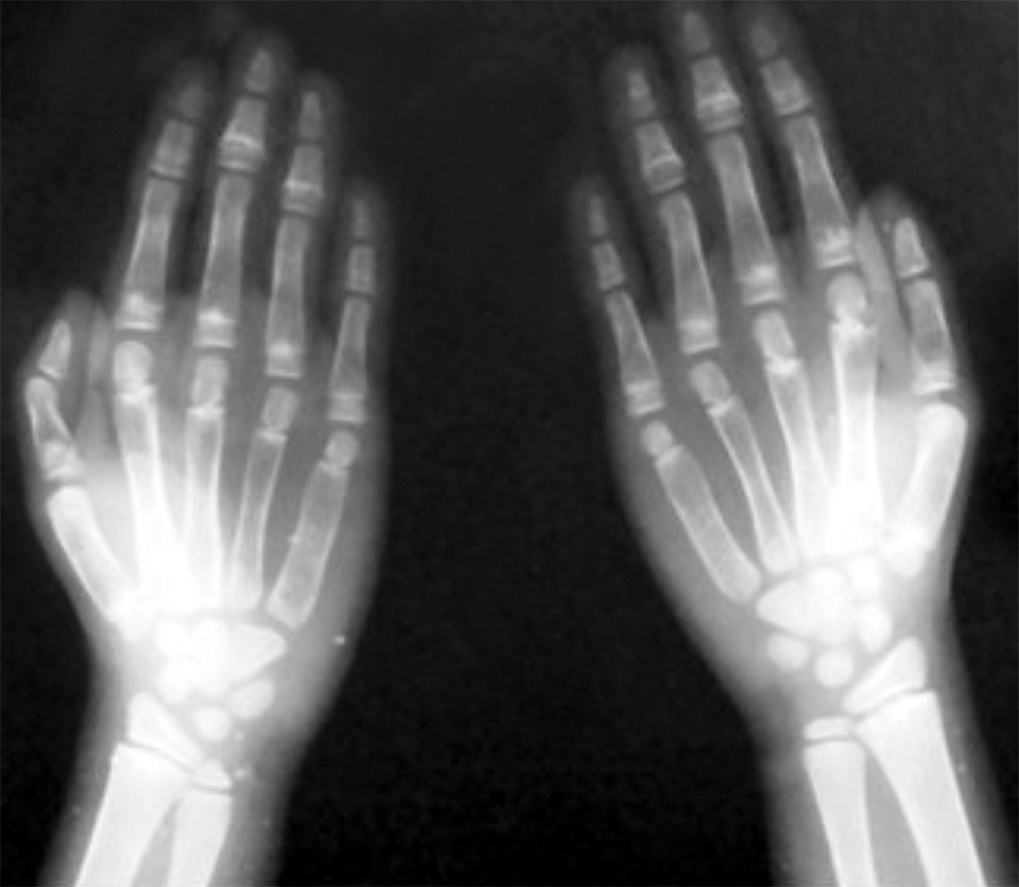
Hand wrist radiograph showing hypoplastic phalanges

The diagnosis of cleidocranial dysplasia was confirmed radiographically further by obtaining X-ray skull (AP and lateral) (Figs 14 and 15) and X-ray hand wrist (Fig. 16).

## DISCUSSION

It has been a relatively under-diagnosed entity and due to its clinical features can be mistaken for a number of other conditions (e.g. Noonan syndrome, Turner’s syndrome, hypothyroidism and other skeletal dysplasia), before the diagnosis is confirmed radiologically.^7^

Treatment options^8^ for the oral manifestations have included practices such as vitamins and thyroid extracts, full dentures postextraction (now not recommended), over-dentures, endosseous implants and iliac bone grafts to increase retention of maxillary dentures. Dentists have also tried to aid self-eruption by extraction of supernumerary and retained primary teeth when the permanent teeth develop at least 2/3rds of the roots. Another option has been to simultaneously extract all the deciduous supernumerary and retained teeth after the permanent 6 erupt and give a space maintainer appliance and either to wait for the teeth to erupt on their own or to use orthodontic traction forces to aid eruption, postexposure of the teeth. Early extraction of primary teeth and cutting bony windows to allow the permanent to erupt has been advocated to allow for eruption without orthodontic treatment.

## CONCLUSION

Though the people suffering from cleidocranial dysplasia can and do lead largely normal lives, they may suffer a lot through society. Besides the dental and skeletal handicap and the hearing deficit, there is considerable psychological trauma associated with the condition. To treat such a patient an interdisciplinary holistic approach is needed with Dentists (Pedodontics, oral surgeons and orthodontists), ENT surgeons, orthopedic surgeons, psychologists, physiotherapists, genetic counselors and often teachers working in tandem.

In our case, the mother had a more pronounced phe-notypic manifestation of the disease in that her apposition of shoulders and deformation of skull and hand shape was more pronounced. She wore over dentures to camouflage her dental condition and suffered from feelings of inferiority and self blame for having passed on the trait on to her son.

She was insistent that her son should not suffer as she had done and wanted definitive dental treatment for him and wanted him to get well so he would not pass on the trait to his progeny.

They were counseled as to the various aspects of the condition and the dental treatment options.

For definitive dental treatment, the serial extraction of deciduous teeth and aiding eruption via application of orthodontic traction forces was decided upon.
